# Bridging the Gap in the Mechanistic Understanding of Electrocatalysis via In Situ Characterizations

**DOI:** 10.1016/j.isci.2020.101776

**Published:** 2020-11-05

**Authors:** Arnav S. Malkani, Jacob Anibal, Xiaoxia Chang, Bingjun Xu

**Affiliations:** 1 Center for Catalytic Science and Technology, Department of Chemical and Biomolecular Engineering, University of Delaware, 150 Academy Street, Newark, DE 19716, USA; 2 College of Chemistry and Molecular Engineering, Peking University, Beijing 100871, China

**Keywords:** Chemistry, Electrochemistry, Electrochemical Energy Conversion, Surface Chemistry, Energy Materials

## Abstract

Electrocatalysis offers a promising strategy to take advantage of the increasingly available and affordable renewable energy for the sustainable production of fuels and chemicals. Attaining this promise requires a molecular level insight of the electrical interface that can be used to tailor the selectivity of electrocatalysts. Addressing this selectivity challenge remains one of the most important areas in modern electrocatalytic research. In this Perspective, we focus on the use of in situ techniques to bridge the gap in the fundamental understanding of electrocatalytic processes. We begin with a brief discussion of traditional electrochemical techniques, ex situ measurements and in silico analysis. Subsequently, we discuss the utility and limitations of in situ methodologies, with a focus on vibrational spectroscopies. We then end by looking ahead toward promising new areas for the application of in situ techniques and improvements to current methods.

## Graphical Abstract

**Figure:**
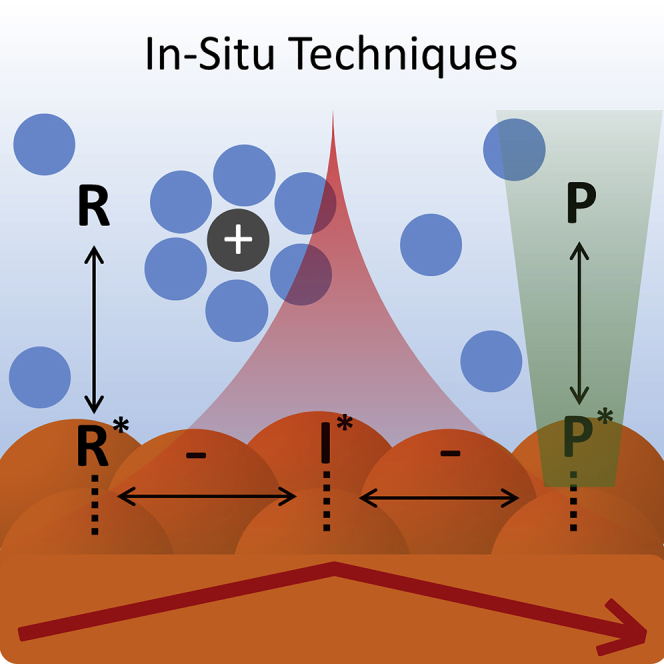

## Introduction

With renewable electricity increasingly available and affordable (Haegel et al., 2017), electrocatalytic processes are expected to play a key role in the future energy and chemical landscape (De Luna et al., 2019). The ability of electrochemical systems to facilitate scaling-out, i.e., deployment in a distributed fashion, rather than scaling-up, i.e., deployment in a centralized manner, makes them highly compatible with dispersed renewable-energy sources, such as solar and wind power. However, electrochemical processes remain relatively undeveloped, often due to poor selectivity and energy efficiency. Development of selective and efficient electrochemical processes requires advances in both catalyst and device/process design. This Perspective focuses on the former, with an emphasis on solid electrocatalysts (heterogeneous electrocatalysis), rather than molecular ones (homogeneous electrocatalysis). We note that the boundary between these two sub-branches of electrocatalysis has become increasingly blurred by hybrid strategies, such as grafting molecular catalysts onto conducting supports (Willkomm et al., 2019), so that much of the discussion may apply to both. Like the more mature thermocatalysis, electrocatalysis is propelled by the twin engines of fundamental studies and application-driven engineering research. While the former utilizes well-defined materials and conditions to extract reliable structure-activity relations (Kuzume et al., 2007; Marković et al., 1994; Schouten et al., 2012), the latter seeks to achieve performance targets, such as current density and energy efficiency, by taking advantage of the entire accessible composition and structural parameter space (Jeon et al., 2018; Raciti et al., 2017; Yang et al., 2020). The difference in focus of the two approaches can create friction in the catalyst development process. On the one hand, principles extracted from idealized model systems may prove insufficient to guide the design of electrocatalysts with complex compositions and structures. On the other hand, the underpinnings of high-performance catalysts (often with elaborate compositions and structures) are frequently insufficiently understood to inform further catalyst design. In this Perspective, we discuss how deficiencies in widely employed workhorse experimental techniques in evaluating, characterizing and understanding electrocatalysts lead to this disconnect, and how existing and future advances in in situ/operando characterization techniques may help bridge this gap in understanding and accelerating the development of electrocatalysts. We note that “in situ” and “operando” techniques in catalysis have an important distinction, i.e., “operando” refers to characterizations that are conducted while reactivity is simultaneously measured, while “in situ” only requires that characterizations are carried out at conditions close or identical to reaction (Bañares, 2005). In the context of this Perspective, we do not intend to highlight this distinction and refer to both as “in situ” below.

## Common Methods in Heterogeneous Electrocatalysis

### Activity and Selectivity Evaluations

Cyclic voltammetry (CV) and electrochemical reactivity tests represent the most widely used techniques in assessing the performance of electrocatalysts. Accurate determination of both activity (overall rates) and selectivity are critical in evaluating the effectiveness of electrocatalysts. Selectivity plays an equally, if not more important role than activity in electrocatalysis, as overpotential may, to an extent, drive higher rates, but selectivity depends strongly on the properties of the electrocatalyst. For electrochemical processes without strong competing reactions, CV allows for preliminary activity evaluations of electrocatalysts. This method works well for many organic electrochemical reactions, particularly in organic solvents (Baizer and Lund, 1983), but proves less informative for aqueous systems, in which strong hydrogen or oxygen evolution activity often obscures CV features corresponding to reactions of interest (Candido and Gomes, 2011; Le Duff et al., 2017). Additionally, competitive adsorption may cause cathodic shifts in CV peaks for mixed reactant systems compared to the individual species (Anibal and Xu, 2020; Ardizzone et al., 2002), leading to difficulty in interpreting voltammograms. For these reasons, reactivity tests in batch or flow reactors with ex situ chemical quantifications, e.g., nuclear magnetic resonance (NMR) and gas chromatography (GC), are often preferred over CVs for selectivity evaluation. These reactivity tests, and associated analytical techniques, have been extensively used for aqueous systems with strong hydrogen evolution activity, such as the CO_2_ reduction reaction (CO_2_RR) and CO reduction reaction (CORR) (Hori, 2008; Wang et al., 2018). While most reactivity studies focus on the potential dependence, other electrochemical parameters, such as catalyst facets (Huang et al., 2017; Luc et al., 2019; Raciti et al., 2017; Roberts et al., 2015) and the electrolyte cation (Malkani et al., 2020c; Pérez-Gallent et al., 2017b; Resasco et al., 2017; Singh et al., 2016) have also been varied to investigate their effects on reaction selectivity. Reactivity tests have also been applied to various organic electrochemical systems, with recent activity focused on the reduction of biomass species (Baizer and Lund, 1983, Andrews et al., 2020; Lopez-Ruiz et al., 2019; Song et al., 2018). Generally, reactivity tests show the impact of a given electrochemical parameter on the overall rate and selectivity, but, in most cases do not provide mechanistic information to explain the observed impact. The complex and interconnected nature of interfacial species make it difficult to elucidate the precise effect of altering an electrochemical parameter (Malkani et al., 2020c). Modifying one experimental variable may trigger a multitude of changes at the electrochemical interface, making it difficult to attribute changes in reactivity or selectivity to a specific cause. The impact of potential on the CO_2_RR product distribution provides an example. In this case, changing the applied potential affects both intrinsic rates and the interfacial pH. For optimal efficiency, the CO_2_RR typically occurs in near neutral electrolytes, e.g., sodium bicarbonate solution (Jouny et al., 2019a). At large overpotentials, the strong reduction rates increase the pH near the surface, altering the local CO_2_ concentration and, consequently, product distributions (Dunwell et al., 2018; Hori et al., 1989). This convolution of potential and interfacial pH precludes a clear understanding of the effect of potential on selectivity without in situ characterization techniques capable of decoupling these effects. Reactivity trends with other experimental parameters are likely similarly convoluted. This complexity makes reactivity data alone insufficient for understanding selectivity trends and reaction mechanisms.

### Ex Situ Characterizations

Ex situ characterization of prepared catalysts typically provides a starting point for understanding catalytic materials but not a complete picture. Electrocatalytic research routinely employs ex situ characterizations, such as scanning/transmission electron microscopy, physi/chemisorption and X-ray photoelectron spectroscopy, as well as electrochemical methods, such as CV and capacitance, to characterize catalysts before and after reaction. These techniques allow confirmation of catalyst composition or structure and, in some cases, can provide quantitative information, such as surface area or particle size. Often ex situ measurements are also employed to understand catalyst selectivity or activity, by correlating catalyst structure with reactivity data. Unfortunately, correlating ex situ measurements with trends in selectivity or activity may prove misleading, as catalysts can, and often do, change under reaction conditions (Kim et al., 2014; Zhao et al., 2020). This concern appears especially acute in electrocatalysis for three reasons: (1) electrocatalytic reactions are intrinsically redox reactions, which tend to change the oxidation state of the catalyst (Jiang et al., 2018); (2) no widely applicable ex situ titration method exists for active sites in electrocatalysis, as certain redox sites may only manifest under reaction conditions (Dong et al., 2019; Malkani et al., 2019; Zhao et al., 2020), e.g., potential; and (3) many electrocatalysts are prepared by nonelectrochemical methods (Dunwell et al., 2017a; Li et al., 2019a; Wang et al., 2018), such that potential and current represent stress factors not experienced during synthesis and may cause structural changes. Combined, these considerations make it difficult to accurately estimate in situ properties by ex situ means. This difficulty makes in situ characterization a critical complement to ex situ techniques to establish reliable structure-activity relations. Further, the more reliable in situ characterizations also provide representative models to assist in silico electrocatalytic investigations.

### In Silico Investigations

**Scheme 1:**
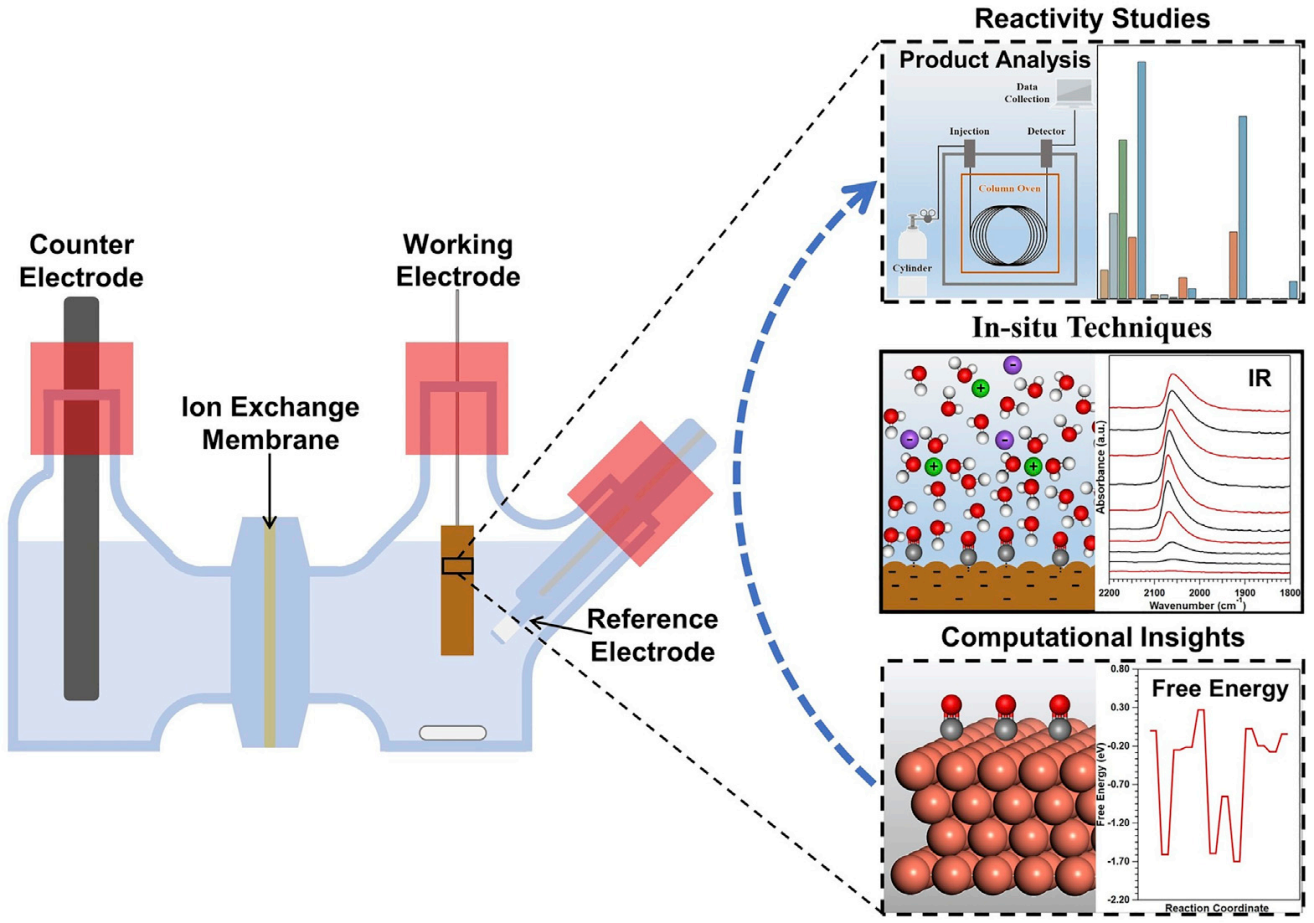
Schematic Depicting the Role of In Situ Characterization Techniques They are a bridge between reactivity and computational studies.

Rapid improvements in both the accuracy and availability of modern electronic structure calculations have made in silico investigations an indispensable component of electrocatalytic research. Electronic structure calculations, such as those based on density functional theory, can provide: (1) guiding principles for catalyst design by identifying key properties or descriptors of catalytic materials, such as in “d-band theory” (Zhao et al., 2019); and (2) molecular level mechanistic information typically inaccessible to experimental methods, e.g., detailed reaction pathways or the structures of activated complexes. Predictions based on the “d-band theory” and other descriptors, such as binding energies, have been repeatedly verified experimentally (Lima et al., 2007; Xu et al., 2020), demonstrating their reliability. Despite this general effectiveness, the representativeness of the computational model generally limits the accuracy of calculations for a given system. These limitations typically stem from three main factors, (1) the maximum number of atoms incorporated in the model; (2) the ability to accurately model key parameters in electrocatalytic systems, e.g., electric potential, solvent molecules and ions; and (3) the knowledge of atomic or molecular level structural information for the experimental system. The first limitation has become increasingly alleviated as computational resource becomes more abundant. The second challenge falls on practicing computational electrochemical researchers, which falls outside the scope of this experimental technique-focused Perspective. On the third front, in situ methods can play an important role. Experimental insights gained from in situ techniques can help bridge the gap between information accessible through reactivity and ex situ characterization techniques, and that required to construct accurate and representative computational models to achieve a molecular level understanding of reactions at electrochemical interfaces (Scheme 1).

## Real-Time and In Situ Techniques

In this section, we discuss a selected group of real-time and in situ techniques that can provide the much-needed mechanistic insights to further the understanding of electrocatalysis.

### Gaining Mechanistic Insights with Advanced Product Analysis Methods

Mechanistic insight can be obtained by modifying reactivity tests through techniques such as isotopic labeling. Although the analysis in this method occurs ex-situ, isotopic labeling provides in situ insights by preserving mechanistic information (Lin et al., 2020). In this technique, key atoms in the reactants are “labeled” with isotopes (without changing their reactivity) and then are monitored throughout the reaction to help elucidate intermediate species and reaction mechanism. Jouny et al. used C^18^O to elucidate the reaction intermediate for the co-electrolysis of CO and NH_3_ to acetamide (Jouny et al., 2019b). They observed ^18^O-labeled acetamide as the dominant product, and suggested that the acetamide was formed through the nucleophilic attack of NH_3_ on a ketene-like intermediate (Jouny et al., 2019b). Chang et al. employed isotopically labeled CO (^13^CO) and acetaldehyde (CD_3_CDO) to determine the C-C coupling pathway for 1-propanol in the CORR. Their results suggested that CO attacks the carbonyl carbon in acetaldehyde during the cross-coupling between them and ends up being hydrogenated to the hydroxymethyl group (-CH_2_OH) in 1-propanol (Chang et al., 2020).

Differential electrochemical mass spectrometry (DEMS) is a technique allowing for real-time analysis of volatile products in electrocatalytic reactions. Although variations of DEMS are typically not considered in situ, they do provide mechanistic insights inaccessible to the conventional techniques such as GC and NMR. Clark et al. used a DEMS setup to study the CO_2_RR on Cu and showed that the concentration of aldehydes relative to alcohols in the vicinity of the cathode was higher than in the bulk electrolyte (Clark and Bell, 2018). This observation suggested that aldehydes produced near the surface undergo further reduction to alcohols before diffusing into the bulk electrolyte (Clark and Bell, 2018). The ability of DEMS to readily differentiate isotopically labeled species also makes a coupling of DEMS and isotopic labeling attractive (Hasa et al., 2020; Wang et al., 2019b). Neither technique can directly identify active sites or provide other surface insights, but such information can be inferred from changes in reaction pathways or reactive intermediates and in conjunction with the other techniques discussed below.

### In Situ Spectroscopic Techniques

**Scheme 2:**
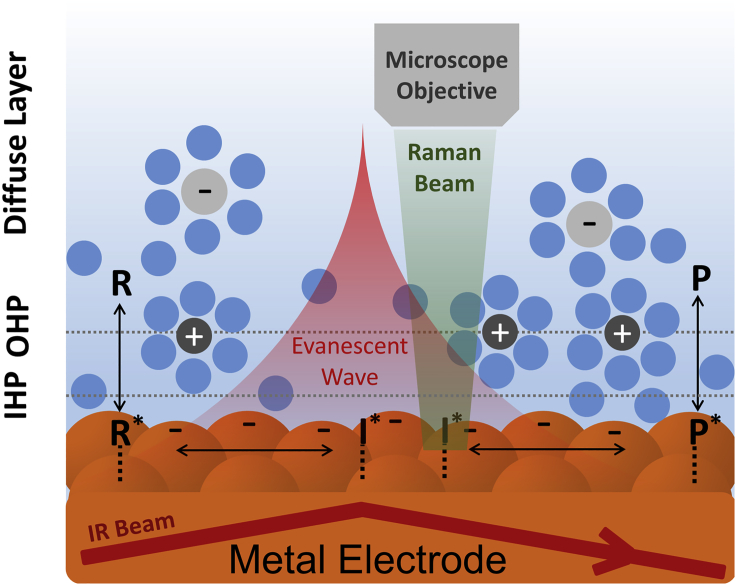
Schematic of the Electrochemical Interface in SEIRAS and SERS Experiments IHP and OHP stand for the inner Helmholtz plane and the outer Helmholtz plane, respectively. R and P represent reactant and product, respectively. R^∗^, I^∗^, and P^∗^ stand for adsorbed reactant, intermediate and product, respectively.

In situ spectroscopies can provide detailed molecular information for species within the double layer (inner Helmholtz plane [IHP] and outer Helmholtz plane [OHP], respectively, in Scheme 2) under reaction conditions and can complement macroscopic information obtained from conventional electrochemical techniques. In situ X-ray absorption spectroscopy (XAS) can probe the oxidation state and coordination environment of metal atoms at, or near, reaction conditions, allowing for more accurate structure-selectivity relationships than ex situ methods. The main limitation of this technique comes in surface sensitivity, as XAS probes bulk, as well as surface, atoms, and bulk signals may overwhelm those from the surface (Wang et al., 2019a). Fortunately, this lack of surface sensitivity represents a lesser concern for catalysts with small metal clusters or single atom active sites, as most or all metal atoms are on the surface. Mukerjee et al. investigated the effect of Pt particle size (30–90 Å) on oxygen reduction using in situ XAS and showed that as the particle size was reduced to below 50 Å, the adsorption strength of H, OH and C_1_ compounds such as CO increased (Mukerjee and McBreen, 1998). Yang et al. recently used in situ XAS to suggest oxynitride as the active phase for electrochemical nitrogen reduction on vanadium nitride by correlating the disappearance of the oxynitride band with catalyst deactivation (Yang et al., 2018). The discrimination of X-ray based techniques against lighter atoms generally makes XAS insensitive to most species in the electric double layer, e.g., water and organic species. For these species, surface-enhanced vibrational spectroscopies, i.e., surface-enhanced infrared absorption spectroscopy (SEIRAS) and surface-enhanced Raman spectroscopy (SERS), prove more informative because of their high interfacial sensitivity (Hatta et al., 1982; Li et al., 2010; Osawa, 2001; Toporski et al., 2018). The strong IR adsorption of water can present a challenge for aqueous electrochemical systems. However, the use of a Kretschmann attenuated total reflection (ATR) configuration largely mitigates this issue and ATR-SEIRAS has become an increasingly popular, and powerful, technique in modern spectroelectrochemistry (Kas et al., 2019; Wain and O'connell, 2017). In ATR-SEIRAS, a thin metal catalyst layer is deposited onto a Si (or Ge) ATR crystal to act as the working electrode. An IR beam internally reflects off the Si/metal interface, creating an evanescent wave which penetrates into the solution (Scheme 2). Importantly, the metal layer enhances the evanescent wave, but only near the catalyst surface, effectively limiting signal intensity to within 5–10 nm from the catalyst surface and making SEIRAS surface sensitive (Kas et al., 2019; Osawa, 2001). SERS, the Raman complement to SEIRAS (Scheme 2), also shows surface enhancement. Although the SERS enhancement originates from the short range surface plasmonic effects, with significant enhancement only for coinage metals (Cu, Ag, and Au) (Sharma et al., 2012; Willets and Van Duyne, 2007). Enhancement on non-coinage metals can occur using shell-isolated nanoparticle-enhanced Raman spectroscopy, in which SiO_2_ coated Au or Ag particles are added to create local signal enhancement (Li et al., 2010; Toporski et al., 2018). Generally, SEIRAS and SERS provide complementary information in terms of accessible vibrational modes and the spectral window. They do, however, show differences in their spatial and temporal resolutions. SERS is known to possess an enhanced spatial resolution owing to the narrow, micron-sized incoming laser beam that can detect variations in signal intensity at different spots on a heterogeneous surface (Zhao et al., 2020). This feature can be useful in studying parts of a catalyst surface in isolation; an aspect that is not possible with SEIRAS. Unfortunately, the SERS signal is limited by the weak Raman scattering effect and thus spectrum collection in SERS requires longer acquisition time than SEIRAS, making it less suitable in detecting short-lived reaction intermediates. Water is a strong absorber of infrared light but a relatively weak scatterer of Raman signal (Wain and O'connell, 2017). Thus, if the vibrational modes of water in the electrolyte are the subject of study, SEIRAS can be a useful technique to employ. SERS, on the other hand, can be used to avoid the interference of water's spectral features. Despite these differences, the high surface sensitivity of both methods can provide various molecular level insights into the electrochemical interface. Below we discuss a few unique insights available from in situ vibrational spectroscopies.

### Identifying near Surface Species

The surface sensitivity of SERS and SEIRAS makes them potent tools for identifying surface species. The observation of IR or Raman bands allows for the identification of surface species, e.g., CuO_*x*_(OH)_*y*_ (Zhao et al., 2020) and OH (Dong et al., 2019; He et al., 2020), adsorbates, e.g., adsorbed CO (Gunathunge et al., 2017; Wuttig et al., 2016), and interfacial, but nonspecifically adsorbed species, e.g., ions (Dunwell et al., 2017b; Li et al., 2020; Malkani et al., 2020c; Yang et al., 2019). Typically, a series of spectra are collected while varying an experimental parameter, e.g., potential or time, to highlight changes in spectral features. Species identification informs the development of mechanistic hypotheses, reaction pathways and rate limiting steps. Generally, SEIRAS and SERS prove most effective for reactions with relatively stable and strongly adsorbed intermediates, such as larger organic molecules in non-aqueous solvents and CO on metals. For example, in situ IR spectroscopy has been used to identify ketyl radical intermediates for the reduction of larger aldehydes and ketones in organic solvents (Bewick et al., 1996; Pons et al., 1983; Tallant and Evans, 1969), and more recent work has extended these spectroscopic investigations of ketones to the aqueous phase (Anibal et al., 2020; Anibal and Xu, 2020). Spectroscopic identification also faces limitations in peak assignment. Although peaks may be confirmed as intermediates using control experiments or by changing potential, peak assignment to specific species often remains ambiguous. An observed peak might reasonably be assigned to multiple proposed intermediates with similar functional groups. For example, it remains unclear if the observed ketyl radicals in ketone reduction correspond to protonated or unprotonated radicals (Anibal et al., 2020; Bewick et al., 1996; Pons et al., 1983; Tallant and Evans, 1969). Further, the lack of IR or Raman standards for unstable intermediates, such as radicals, creates difficulty in establishing even an expected frequency range for these species. In situ identification may also prove limited for reactions with unstable intermediates, such as in the CO_2_RR and CORR. Adsorbed CO in most cases appears as the only detectable CO_2_RR and CORR intermediate using in situ IR spectroscopy (Dunwell et al., 2017a; Wuttig et al., 2016), although other intermediates, such as CO dimers (Pérez-Gallent et al., 2017a) and COOH (Dunwell et al., 2017c), are occasionally claimed.

**Figure 1:**
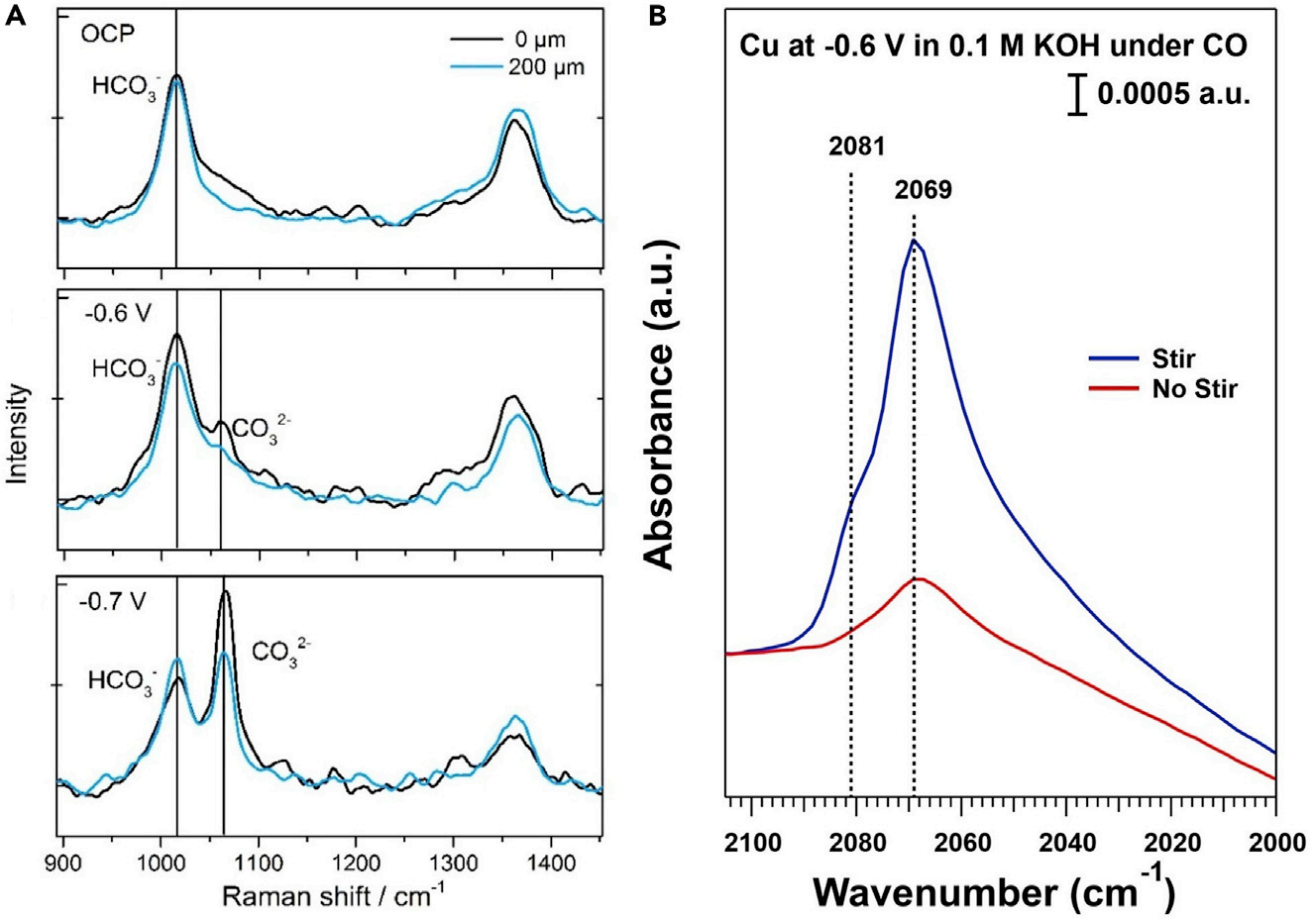
In Situ Surface-Enhanced Vibrational Spectroscopies Identifying Near Surface Species (A) SER spectra on Cu at different potentials in 0.1 M KHCO_3_ collected directly on the surface (0 μm; black line) and 200 μm away from the surface (blue line). Figure adapted from (Klingan et al., 2018) with permission. (B) SEIRA spectra for CO on Cu under stirring and non-stirring conditions. Figure adapted from (Malkani et al., 2020b) with permission.

Surface-enhanced vibrational spectroscopies can also provide insights into the relative concentration of electroactive species at a catalyst surface. (Semi)Quantitative analysis with SEIRAS (Dunwell et al., 2018) and SERS (Jiang et al., 2019) can prove informative but needs to be treated with care. For example, surface to surface variation and film damage in SEIRAS can have a significant impact on signal intensity, making it imperative to use the same film/surface or normalize the peak area to a known band. Fortunately, recent modifications to SEIRAS cells by employing a flow configuration allow easier collection of spectra for multiple electrolyte solutions on the same film with minimal film damage (Chen et al., 2006; Malkani et al., 2020a). The ability to measure relative surface concentrations in situ presents many electrochemical applications. For example, SEIRAS and SERS have been utilized to estimate surface pH (Dunwell et al., 2018; Klingan et al., 2018). Klingan et al. used SERS to quantify the increase in interfacial pH at CO_2_RR active potentials (−0.6 and −0.7 V vs. RHE) compared to the open circuit potential by tracking the ratio of the interfacial carbonate and bicarbonate bands (Figure 1A). The spectroscopically observed increase in the surface pH provided an explanation for the increase in selectivity for the HER over the CO_2_RR at high overpotentials (Singh et al., 2016), linking macroscale selectivity trends with microscale phenomena. Spectroscopically determined surface concentrations also provide insight into mass transport. Malkani et al. showed that the loss of forced convection decreased the concentration of linearly bonded CO on Cu terrace (2069 cm^−1^) and step sites (2081 cm^−1^) by ~70% under the CORR conditions (−0.6 V vs RHE, pH ~13) (Figure 1B) (Malkani et al., 2020b). The decrease does not coincide with an IR peak shift, however, suggesting minimal microscale concentration changes despite the lower macroscale coverage. This insight, combined with a negligible effect of stir rate on selectivity, led to the hypothesis that CO adsorbs in patches on the Cu surface (Malkani et al., 2020b). The ability to probe interfacial carbonate, bicarbonate, and surface-adsorbed CO concentrations highlights the potential of surface-enhanced vibrational spectroscopies to track species in the electric double layer that participate or impact surface-mediated electrocatalytic processes.

### Probing the Electrochemical Interface

**Figure 2:**
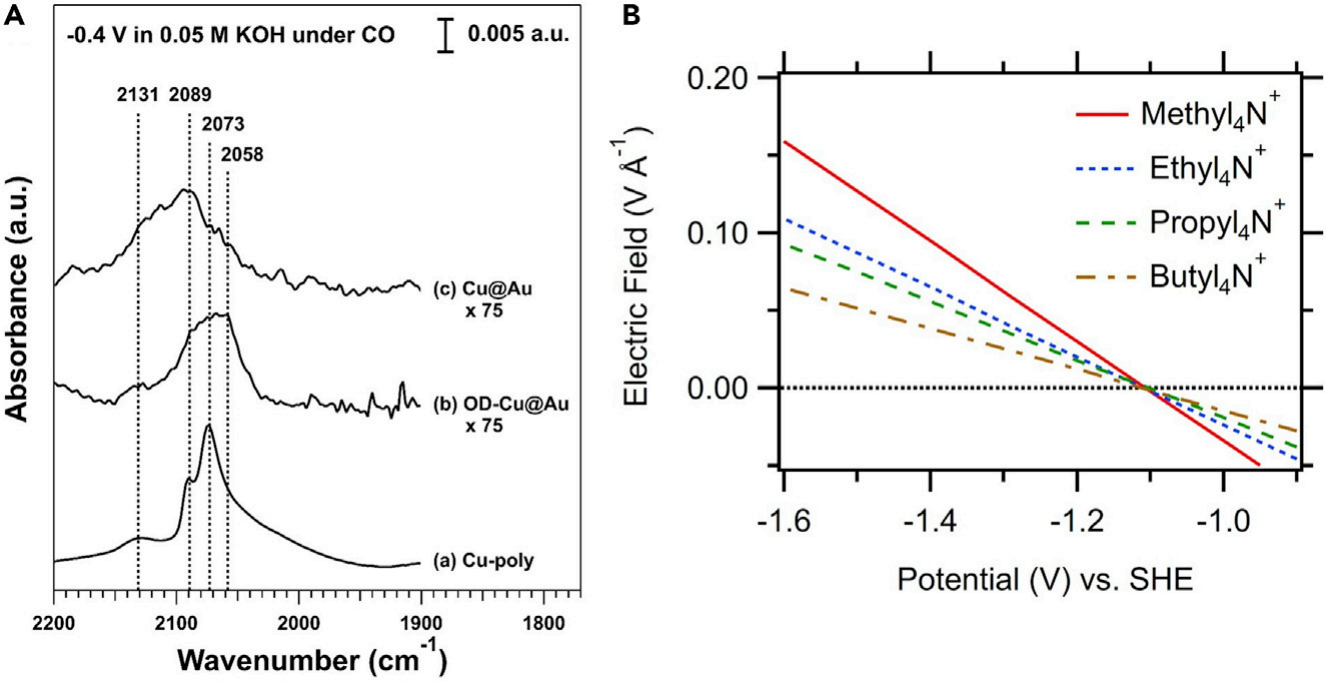
In Situ Surface-Enhanced Vibrational Spectroscopy Probing the Electrochemical Interface (A) SEIRA spectra showing linearly bonded CO adsorbed on different Cu surfaces in CO saturated 0.05 M KOH at −0.4 V vs. RHE. Figure adapted from (Malkani et al., 2019) with permission. (B) The effect of organic cations on the electric field strength within the electrochemical double layer. The electrolyte consisted of 0.1 M alkyl-ammonium hydroxide (pH 9.5). Electric fields were determined from Stark tuning rates. Figure adapted from (Li et al., 2019a) with permission.

In situ vibrational spectroscopies can also probe binding sites available on electrocatalysts under reaction conditions. In many cases, this requires the use of a probe molecule, particularly for SEIRAS, due to its limited spectral window. The Si crystal commonly employed in SEIRAS show strong IR absorption below 1200 cm^−1^ (Zhu et al., 2019), making adsorbate-catalyst vibrations, such as metal-C and metal-O, inaccessible. Although recent work using micro-machined Si wafers as reflection elements has shown effectiveness in mitigating this difficulty (Morhart et al., 2017). Typically, molecules with both strong binding and IR absorption, such as CO, serve as probes in infrared investigations. For the CO_2_RR and CORR, adsorbed CO has the added benefit of being a reaction intermediate (Wuttig et al., 2016). Gunathunge et al. used CO as a probe to study the reconstruction of Cu surfaces under CO_2_RR conditions in bicarbonate electrolytes (Gunathunge et al., 2017). Based on a ~30 cm^−1^ blueshift in the linearly bonded CO band, they suggested that the Cu surface reconstructed at more cathodic potentials to expose undercoordinated defect sites. Vibrational spectroscopies can also elucidate the types of active sites occupied under reduction conditions, which often impact selectivity. For example, defect (step and kink) sites have been shown to enhance the C-C coupling pathway in the CO_2_RR (Wang et al., 2018). Observing changes in the available sites among a series of catalysts can help explain selectivity trends. Malkani et al. used operando SEIRAS to track the adsorbed CO bands at −0.4 V vs. RHE for Cu catalysts prepared in different ways (Malkani et al., 2019). They observed similar CO band positions for polycrystalline Cu (Cu-poly) and Cu micron particles (Cu@Au) but noticed a lower wavenumber (2058 cm^−1^) CO band on oxide derived Cu (OD-Cu) (Figure 2A) (Malkani et al., 2019) corresponding to the Cu (100) facet observed on single crystals (Hori et al., 1998). The observation of the Cu(100) facet, previously shown to favor C-C coupling to ethylene (Roberts et al., 2015), helped explain the improved C-C coupling selectivity for OD-Cu at low overpotentials (Malkani et al., 2019). CO has also been employed as a probe molecule to investigate the impact of other interfacial species, e.g., cations in the electrolyte at the electrochemical interface. Dunwell et al. showed that the ratio of bridge bonded to linearly bonded CO on Pt depends on both the potential and the electrolyte cation (Dunwell et al., 2017b). SERS also shows utility in elucidating surface sites. The wider spectral range (as low as 100 cm^−1^) makes in situ SERS less reliant on probe molecules and allows the observation of C-metal and O-metal vibrations which can help elucidate catalyst oxidation state or speciation. The speciation of the catalyst surface represents an important, and often debated, parameter for many reaction systems. For example, the speciation of the Cu surface in the CO_2_RR and CORR has long been a topic of discussion (Deng et al., 2016; Lum and Ager, 2018; Mistry et al., 2016; Ren et al., 2015; Zhao et al., 2020). Due to the high oxophilicity of Cu, air exposure typically leads to the formation of oxide and hydroxide species on the surface and it remains ambiguous if these species persist under reduction conditions. SERS provides one means of probing these species. Recently, Zhao et al. reported that Raman bands attributable to CuO_*x*_(OH)_*y*_ and Cu-O_ad_ species were observed at as low as −0.8 V vs. RHE under CORR conditions (Zhao et al., 2020). Cu hydroxide species were also observed at reducing potentials during the co-electrolysis of CO_2_ with O_2_ (He et al., 2020). In addition, SERS has also been employed to identify the active phase in other electrocatalytic reactions such as the oxygen reduction reaction and the hydrogen evolution reaction (Chen et al., 2020; Dong et al., 2019; Wang et al., 2019c).

Less common than species or site identification, electric field strength can also be probed by in situ vibrational spectroscopy using the vibrational Stark effect. First extended to electrochemical systems by Lambert (Lambert, 1988), Stark tuning refers to the shift of the peak position of an adsorbate with applied potential, which results from the interaction of the vibrational dipole moment with an applied electric field. For electrochemical systems, Stark tuning generally occurs within the OHP (Scheme 2), 3-5 Å from the surface (Lambert, 1988), as only this region contains the strong electric field required. This condition makes Stark tuning sensitive to adsorption distance, such that species with weaker binding outside the OHP are expected to show little to no Stark tuning. Anibal et al. observed such a lack of Stark tuning for the reduction intermediates of benzaldehyde and furfural (Anibal et al., 2020; Anibal and Xu, 2020). For species adsorbing within the IHP (Scheme 2), such as adsorbed CO, the Stark tuning rate allows quantitative estimation of the electric field at the interface (Lambert, 1988). We note that the estimated field represents the component of the electric field parallel to the dipole moment and thus can depend on adsorbate orientation. However, this correction appears small for linear adsorbates normal to the surface, such as CO, and the methodology generally produces reasonable estimates of electric field strength (Lambert, 1988; Li et al., 2019a; Roth and Weaver, 1992). Importantly, these estimates do come with another caveat. The peak position of adsorbates also depends on their surface coverage (Sartin et al., 2018), so that Stark tuning measurements are only reliable at a constant coverage. Stark tuning has been employed effectively to estimate electric fields. Li et al. used Stark tuning of linearly bonded CO to estimate the electric field during CO reduction for different organic cations (Li et al., 2019a) (Figure 2B). This quantitative, in situ measurement of electric field represents an important tool for investigating selectivity. Similar measurements in nonaqueous electrolytes were made earlier by Roth and Weaver on Pt (Roth and Weaver, 1992). Electric field strength has been computationally suggested as an important parameter in controlling selectivity for reactions such as CO reduction (Ringe et al., 2019). A stronger electric field has been suggested to stabilize key reaction intermediates, improving the energetics of the C-C coupling rate determining step (Resasco et al., 2017). Although more recent results suggest that factors other than the interfacial electrical field play a significant role in determining the CORR activity and selectivity (Li et al., 2019a; Malkani et al., 2020c). Further in situ measurements of the electric field will allow for verification and refinement of these hypotheses.

## Perspective on Advancing Electrochemical Spectroscopy

### Promising Future Avenues for Spectroscopic Investigations

With the development of nanoscience, alloyed and multicomponent structures have attracted increasing attention in electrocatalysis due to the enhancement of various physical and chemical properties by synergistic effects between metals (Gilroy et al., 2016). However, investigating the origin of performance enhancement in bimetallic systems, such as identifying which metal atoms constitute adsorption sites, remains challenging. In situ spectroscopies can provide insight into bimetallic enhancement by differentiating specific metal-adsorbate interactions (Kas et al., 2019; Toporski et al., 2018). For example, Zhang et al. synthesized an Ag-Cu tandem catalyst for the electroreduction of CO_2_ to methane and employed in situ SEIRAS to probe CO adsorption sites on the catalyst surface (Zhang et al., 2019). The Ag-Cu surface exhibited an intense band for bridge-bonded CO in contrast to the linearly bonded CO bands on Ag and Cu (Zhang et al., 2019), indicating that CO adsorption sites on the Ag-Cu surface were not just a mixture of those on Cu and Ag surfaces (Zhang et al., 2019). A similar observation was also made on bimetallic Cu_0.9_Ni_0.1_ under CORR conditions (Yang et al., 2020). SERS has also been employed to show that an Ag/Cu bimetallic has a wider distribution of CO binding configurations than monometallic Cu, which offers an explanation for its enhanced ethanol selectivity during the CO_2_RR (Li et al., 2019b). Zhong et al. showed that a bimetallic layered conjugated metal organic framework (MOF) catalyst of copper-phthalocyanine (CuN_4_) ligands and zinc-bis (dihydroxy) complex (ZnO_4_) linkages was selective for the electrochemical conversion of CO_2_ to CO (Zhong et al., 2020). They employed in situ XAS to probe the structure of the MOF during reaction conditions and showed that neither catalytic center (CuN_4_ or ZnO_4_) was reduced to its metal form, confirming the stability and activity of the bimetallic MOF (Zhong et al., 2020). These examples represent a growing number of publications demonstrating the potential of in situ spectroscopy to identify active sites on multicomponent catalysts under reaction conditions. Future studies to understand the interfacial electric field near the bimetallic surface using Stark tuning measurements may also improve the understanding of bimetallic activity and selectivity by elucidating the impact of catalyst composition on the local electric field.

The investigation of organic electrochemistry presents another promising area for in situ spectroscopies. The electrochemical oxidation and reduction of organics offers promise both as an industrial synthesis technique (Sequeira and Santos, 2009) and for upgrading raw materials, such as biomass species (Carneiro and Nikolla, 2019). Both processes require effective selectivity control, especially for mixtures with multiple organics, and would benefit greatly from an understanding of both reactive intermediates and relative surface concentrations. In situ IR has been employed for the observation of ketyl radical species (Anibal et al., 2020; Bewick et al., 1996; Pons et al., 1983; Tallant and Evans, 1969) and has been recognized as a means of investigating organic electrochemical reactions (Baizer and Lund, 1983). However, in situ techniques remain relatively underutilized for organics, relative to the CORR and CO_2_RR, despite the greater promise offered by the more stable organic intermediates. In this regard, further investigations into fundamental aspects of organic electrochemistry offer a promising new area in spectro-electrochemistry. Electrocatalytic conversion of organic species does present some difficulties for in situ spectroscopies, mainly those in aqueous systems. The presence of water can interfere with peak identification via convolution with its strong IR peak or by reducing the stability of intermediates, such as radical species. These difficulties may limit the effectiveness of in situ spectroscopy for intermediate identification. An additional difficulty arises in respect to the Stark tuning effect of interfacial organic species, or the lack thereof. Unlike small molecules, such as CO, interfacial organics often do not show appreciable Stark tuning rates. The lack of Stark tuning for organic peaks prevents calculating double layer properties directly and would require the introduction of a trace probe molecule such as CO or thiocyanate. Combined, these challenges limit the effectiveness of in situ spectroscopy for organic electrochemical systems and highlight the need to develop more sensitive and versatile techniques. However, neither poses an insurmountable barrier and organo-electrochemistry offers a promising field for further spectroscopic investigation, particularly in light of the recent interest in electrochemical biomass upgrading (Carneiro and Nikolla, 2019).

### Approaching Fundamental Understanding in Electrocatalysis

To accelerate the progress in achieving molecular level mechanistic understanding in electrocatalysis, further development in in situ characterization techniques is needed to bridge the gap between the macroscopic reactivity and microscopic computational insights. Three general directions appear promising, (1) enhancing the capability of existing techniques; (2) combining complementary techniques for more comprehensive understanding; and (3) developing novel techniques with enhanced sensitivity and spatial/temporal resolution. Development of spectroscopic cells that closely mimic working catalytic conditions represents an important, but often overlooked, direction in obtaining mechanistic insights with existing in situ spectroscopic techniques. Recent work studying the CO reduction has shown that the surface coverage of CO decreases at moderate CO reduction rates (Malkani et al., 2020b). This mass transport effect contributes to changes in reaction selectivity between the CORR and the competing HER. A comparison between reactivity data obtained in a batch (Zhao et al., 2020) and flow cell (Jouny et al., 2018) at −0.6 V vs. RHE using the same Cu MP catalyst shows an improvement in the CORR selectivity from ~5% to ~60% with the improved mass transport in the flow cell. This contrast in selectivity highlights the importance of performing spectroscopic tests at the same conditions as reactivity tests in order to make robust mechanistic claims. This consideration, has rarely been taken into account, even though flow cells have previously been demonstrated for both IR and Raman investigations of electrochemical reactions (Chen et al., 2006; Lu et al., 2020; Nakamura et al., 2007; Zhao et al., 2020). Introduction of on-line mass spectrometers to flow electrolysis cells capable of achieving high current densities in the CO_2_ and CO reduction reactions through flow electrolyzer mass spectrometry (Hasa et al., 2020), is another method to obtain mechanistic information at close to practical electrolytic conditions. Membrane electrode assembly (MEA) configurations have become increasingly popular in electrochemical devices, particularly for fuel cells (Xing et al., 2019), and CO and CO_2_ electrolyzers (Larrazábal et al., 2019), to obtain the high reaction rates necessary for commercialization. The main issue in the application of in situ Raman or IR to MEAs arises in the catalyst support. MEA configurations typically use carbon supports which strongly absorb both visible and IR light, leading to poor signal-to-noise ratios. This high optical absorbance limits the application of IR and Raman to systems which rely on carbon supports, such as shape controlled nanoparticles. Additionally, the support interference makes it prohibitively difficult to investigate differences between MEA and traditional H-cell configurations using IR or Raman. Combined, these issues often limit the applicability of in situ spectroscopy to low current density fundamental systems, i.e. those without carbon supports, creating a friction between fundamental and applied research. Addressing this support limitation will allow for greater utility of IR and Raman in the practical reactivity investigations required for industrialization and scale-up. Fortunately, the challenge does not appear insurmountable, as a number of reports have claimed successful use of Raman (Huguet et al., 2011; Kendrick et al., 2016; Matic et al., 2005) and IR (Fan et al., 1996; Kunimatsu et al., 2010; Sanicharane et al., 2002; Sorte et al., 2016) in an MEA configuration. However, much work is needed to better integrate in situ characterization techniques that typically operate at low current densities into studies with high current density devices for research and diagnostic purposes.

Combining multiple in situ techniques offers another effective strategy to leverage existing methods for a more comprehensive understanding of electrocatalysis. For example, combining in situ IR and Raman spectroscopies (Gao and Weaver, 1989) could help identify surface species and adsorbates, leading to clues for active site identification when correlating with reactivity results. The ability to correlate surface morphology and adsorbate identity during electrocatalytic reactions, e.g., by combining electrochemical scanning probe microscopy and vibrational spectroscopy, could also prove highly informative. For example, tip-enhanced Raman spectroscopy (TERS) is a combination of plasmon-enhanced Raman spectroscopy and scanning probe microscopy which can simultaneously detect both chemical fingerprints and morphological information with a nanometer spatial resolution (Bao et al., 2020; Zeng et al., 2015). Electron paramagnetic resonance (EPR) spectroscopy represents another promising technique for combination with in situ IR or Raman. Probing paramagnetic species, EPR allows detection of intermediates with unpaired electrons, such as carbon radical species. This radical sensitivity may improve the assignment of IR/Raman peaks, particularly for the oxidation or reduction of organic species. In situ EPR has been extensively demonstrated for the detection of radicals in electrochemical systems (Steinberger and Fraenkel, 1964; Tamski et al., 2015; Toybenshlak and Carmieli, 2019; Webster et al., 1998), including carbonyl species (Rieger and Fraenkel, 1962). The technique also appears relatively conducive for combination with in situ IR/Raman, as the large separation in electromagnetic frequencies used in EPR (microwaves) and IR/Raman (infrared and visible, respectively) suggests minimal interference between the methods. In situ vibrational spectroscopy and XAS also represents another promising combination due to their complementary capability. XAS provides a wealth of information about catalyst structure and oxidation state, while vibrational spectroscopies show greater adsorbate sensitivity. Such combined in situ XAS and IR spectroscopies have been effectively used to probe the catalyst in thermochemical systems (Agostini et al., 2018). It should be pointed out that as long as the different in situ techniques probe the same catalytical material at comparable conditions, simultaneous detection by multiple techniques, although generally desired, is not necessarily optimal. In order to allow for concurrent operation, the complexity of the experimental device, e.g., spectro-electrochemical cell, inevitably increases, and design compromises that would make the characterization conditions different from those employed in typical reactivity studies are often needed.

In situ techniques with higher spatial and temporal resolutions could further enhance the mechanistic understanding and accelerate catalyst design. The relatively slow process of establishing an electric double layer upon applying a potential (Atkin et al., 2014; Zwaschka et al., 2019) represents a barrier in understanding transient behaviors in electrocatalytic processes because reactants do not experience an instantaneous potential change (in contrast to reactants in photocatalytic reactions upon absorbing a photon). Thus, the development of novel time-resolved techniques capable of studying transient processes at the electrochemical interface is central to deepening the understanding of how electrocatalytic reactions unfold. The ability to follow electrocatalytic reactions at single molecular level could provide unequivocal evidence for structure-activity relations. Electrochemical TERS has shown promise in this direction (Bao et al., 2020; Pfisterer and Domke, 2018), however, reliably obtaining sufficient resolution to identify surface structure and intermediates on electrode materials at solid-liquid interfaces remains challenging. These current limitations also represent opportunities to advance the understanding of electrocatalytic processes.
